# Supplementary material for: Causal Relationship between Aging and Anorexia Nervosa: A White-Matter-Microstructure-Mediated Mendelian Randomization Analysis
Source: Biomedicines. 2024 Aug 16;12(8):1874. doi: 10.3390/biomedicines12081874 (PMC11351342; doi:10.3390/biomedicines12081874)
Supplement: Supplementary file 1 [file biomedicines-12-01874-s001.zip › Supplementary Figure/Figure S4/Singe SNP plot of MR analysis for TL to ubm-b-1905.pdf]

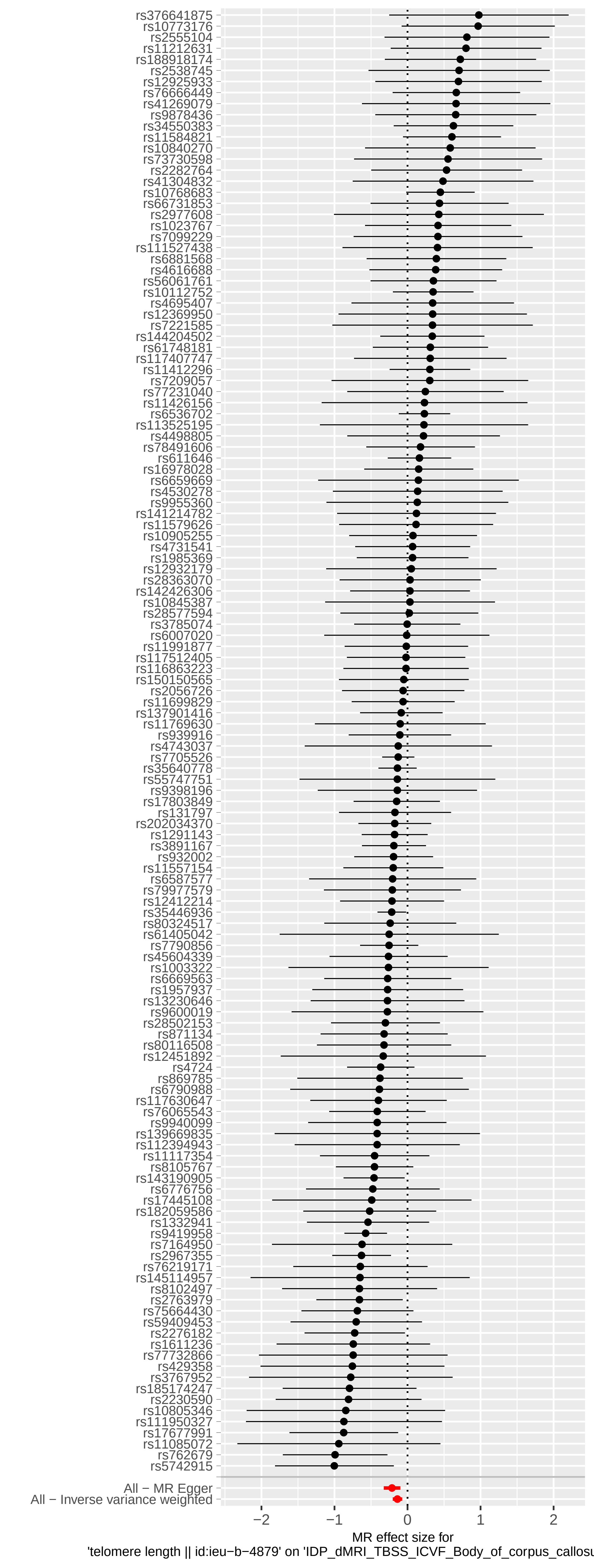
rs376641875
rs10773176
rs2555104
rs11212631
rs188918174
rs2538745
rs12925933
rs76666449
rs41269079
rs9878436
rs34550383
rs11584821
rs10840270
rs73730598
rs2282764
rs41304832
rs10768683
rs66731853
rs2977608
rs1023767
rs7099229
rs111527438
rs6881568
rs4616688
rs56061761
rs10112752
rs4695407
rs12369950
rs7221585
rs144204502
rs61748181
rs117407747
rs11412296
rs7209057
rs77231040
rs11426156
rs6536702
rs113525195
rs4498805
rs78491606
rs611646
rs16978028
rs6659669
rs4530278
rs9955360
rs141214782
rs11579626
rs10905255
rs4731541
rs1985369
rs12932179
rs28363070
rs142426306
rs10845387
rs28577594
rs3785074
rs6007020
rs11991877
rs117512405
rs116863223
rs150150565
rs2056726
rs11699829
rs137901416
rs11769630
rs939916
rs4743037
rs7705526
rs35640778
rs55747751
rs9398196
rs17803849
rs131797
rs202034370
rs1291143
rs3891167
rs932002
rs11557154
rs6587577
rs79977579
rs12412214
rs35446936
rs80324517
rs61405042
rs7790856
rs45604339
rs1003322
rs6669563
rs1957937
rs13230646
rs9600019
rs28502153
rs871134
rs80116508
rs12451892
rs4724
rs869785
rs6790988
rs117630647
rs76065543
rs9940099
rs139669835
rs112394943
rs11117354
rs8105767
rs143190905
rs6776756
rs17445108
rs182059586
rs1332941
rs9419958
rs7164950
rs2967355
rs76219171
rs145114957
rs8102497
rs2763979
rs75664430
rs59409453
rs2276182
rs1611236
rs77732866
rs429358
rs3767952
rs185174247
rs2230590
rs10805346
rs111950327
rs17677991
rs11085072
rs762679
rs5742915
All − MR Egger
All − Inverse variance weighted
−2
−1
0
1
2
MR effect size for
'telomere length || id:ieu−b−4879' on 'IDP_dMRI_TBSS_ICVF_Body_of_corpus_callosu
